# Indoor Localization Method of Personnel Movement Based on Non-Contact Electrostatic Potential Measurements

**DOI:** 10.3390/s22134698

**Published:** 2022-06-22

**Authors:** Menghua Man, Yongqiang Zhang, Guilei Ma, Ziqiang Zhang, Ming Wei

**Affiliations:** 1 National Key Laboratory on Electromagnetic Environment Effects, Shijiazhuang Campus, Army Engineering University, Shijiazhuang 050003, China; manmenghua@126.com (M.M.); zyq@hebust.edu.cn (Y.Z.); mgljyp@163.com (G.M.); 2 School of Information Science and Engineering, Hebei University of Science and Technology, Shijiazhuang 050018, China; zhangzq_work@163.com

**Keywords:** indoor localization, non-contact electrostatic measurements, symbolic regression, sensor compensation

## Abstract

The indoor localization of people is the key to realizing “smart city” applications, such as smart homes, elderly care, and an energy-saving grid. The localization method based on electrostatic information is a passive label-free localization technique with a better balance of localization accuracy, system power consumption, privacy protection, and environmental friendliness. However, the physical information of each actual application scenario is different, resulting in the transfer function from the human electrostatic potential to the sensor signal not being unique, thus limiting the generality of this method. Therefore, this study proposed an indoor localization method based on on-site measured electrostatic signals and symbolic regression machine learning algorithms. A remote, non-contact human electrostatic potential sensor was designed and implemented, and a prototype test system was built. Indoor localization of moving people was achieved in a 5 m × 5 m space with an 80% positioning accuracy and a median error absolute value range of 0.4–0.6 m. This method achieved on-site calibration without requiring physical information about the actual scene. It has the advantages of low computational complexity and only a small amount of training data is required.

## 1. Introduction

The indoor localization of individuals is crucial for “smart city” applications, such as smart homes [1], elderly care monitoring [2], building emergency management [3], occupancy tracking in office spaces [4], smart building controls [5], and energy-saving grids [6]. It is of great value for improving residents’ quality of life [1]. Indoor localization methods can be classified as either “tag-based” or “tag-free” depending on whether the user needs to carry specific hardware devices or not [2]. Tag-based methods have generally utilised signals from WIFI [7,8,9], BLE [10], RFID [11], UWB [12], and LIFI [13]. This technique category was thoroughly examined but has revealed certain limitations in several areas: First, personnel must carry tag devices. Whether it is embedded within a smartphone, watch, bracelet, or an additional specialized device, this will reduce the convenience for the user. Second, each user needs to carry a separate tag. As the number of users increases, the hardware cost and algorithm complexity of the system will increase. Third, tag devices usually require battery power, except for RFID cards. During the charging period of the tag device, the system will not be able to achieve its positioning function. Fourth, various tag devices utilize electromagnetic wave signals as the information carriers. Frequent information interaction is bound to make the electromagnetic environment around the device more severe. Under long-term use conditions, this poses the risk of electromagnetic biological effects, endangering the user’s health.

The preferred indoor localization method is “tag-free” such that users in their home or office environments do not need to carry any specific hardware [2]. Existing tag-free methods rely on a body’s gas [14], sound [15], vibration [16], heat radiation [17], reflection or refraction of light [18], or radio-frequency (RF) signals [19]. However, each of these methods has certain limitations. For example, gas detectors, temperature/humidity sensors, and infrared sensors do not have a high spatial localization accuracy. Camera and microphone arrays present a risk of privacy leakage. Pressure-sensitive or capacitive positioning systems deploy sensors under the floor and are difficult to install or refurbish [20]. Foot-vibration sensors are susceptible to interference from environmental noise. RF location systems based on ultra-wideband radar [21], radio tomography [22], WiFi Doppler [23], and FMCW-modulated radar [24] have high power and cost requirements, and the electromagnetic biological effects caused by actively emitted electromagnetic waves pose a threat to human health. Therefore, studying tag-free indoor localization methods with high accuracy, low power consumption, low cost, good privacy protection, and passivity is of great value.

A comparison between different methods in terms of localization accuracy, power consumption, hardware cost, algorithm complexity, user convenience, privacy protection, and environmental friendliness is shown in Table 1. The localization accuracy is the difference between the actual location and the system output result. The power consumption, hardware cost, and algorithm complexity are calculated in terms of the system as a whole, including the tags and base stations. User convenience concerns whether the user’s device needs routine maintenance, such as charging and calibration. Privacy protection concerns whether there is a risk of a user privacy leakage, such as personal information, images, or voice. Environmental friendliness concerns whether the system will affect the user’s original living environment, such as the additional emission of electromagnetic waves or ultrasonic waves. The “High” or “Low” rating of each indicator is marked relative to the average of the other technologies. The “√” mark means that this is the user’s desired goal. The last row concerns the indoor localization method based on non-contact electrostatic potential measurements that was proposed in this study. It achieved a good balance between these factors. It met the needs of users to the greatest extent possible. Therefore, this approach has more significant application potential.

As is known, electrostatic phenomena caused by the human body are common, for instance, walking on the ground, rising from a chair, rubbing one’s arm against the table, putting on and removing clothing, and other active processes. Rapid contact and separation of the human body from an object will result in the accumulation of static charge on the body, manifested as a fluctuating static potential. Therefore, extensive research has been conducted on personnel sensing and identification technology based on non-contact electrostatic measurements. References [25,26] proposed a high-sensitivity electrostatic induction sensor that enables the recognition of human movements and individual characteristics. References [27,28] used spherical electrode arrays to measure the induced signal from the electrostatic potential of the hand and realized real-time gesture recognition. Prance invented an electrostatic potential sensor (EPS) with high input resistance (~1018 Ω), low input capacitance (~10–17 F), and low noise characteristics in the bandwidth [29]. The commercial version of EPS is known as EPIC (Plessey Semiconductors, PS25255). Reference [30] used EPIC to implement a wearable sensor with orientation and positioning functions. Similarly, a sparse low-power sensor network was implemented based on EPIC. It realized breathing monitoring within 1.5 m by measuring the electrostatic potential of the human body and achieving a 0.1 m location error in a 3 m × 4 m range using four sensors [1]. In [2], an indoor location and human identification system called “platypus” was implemented based on EPIC. A mathematical model for the remote non-contact measurement of human electrostatic potential was proposed based on physical analysis with six sensors arranged within 2 m × 2.5 m. The test results’ normalized root-mean-square error (NRMSE) range was 0.07–0.16 and the location error was 0.16 m. The performance of the above system was mainly affected by the EPIC chip, and the measurement distance and frequency response characteristics of individual sensors could not be flexibly adjusted. The measurement range and accuracy of the system could only be improved by increasing the number and density of sensors. Furthermore, EPIC was discontinued and no replacement models are currently available. To overcome these limitations, this study proposed a new circuit topology for electrostatic potential sensors that can be implemented using commercially available amplifiers. The measurement range and sensitivity of the sensor can be flexibly adjusted. Moreover, it is possible to select amplifiers with different performance parameters according to different needs, thus meeting the requirements of the practical application scenario for sensor performance, power consumption, price, and production cycle.

Under the condition that the physical information of the actual scene (room structure, grounding body distribution, sensor location, etc.) is unknown, the transfer function from the human electrostatic potential to the sensor signal is not unique and the generality of the indoor localization method based on mathematical modeling methods is not good. To address the above problems, a remote, non-contact human electrostatic potential sensor was designed and implemented. An indoor localization method was proposed based on on-site test data and machine learning. This method does not require the usage of the scene’s physical information. After the system is deployed, the human electrostatic potential and motion trajectory are collected simultaneously using electrostatic sensors and an additional high-precision location system. Then, the symbolic regression model is trained using the tested data to obtain the transfer function from the electrostatic sensor signal to the location coordinates. It takes advantage of the low computational complexity of symbolic regression algorithms and the small amount of training data required. After the training, the high-precision localization system is no longer needed.

The main innovations of this study were:

(1) An indoor localization method based on remote, non-contact human electrostatic potential measurement was proposed; this method does not require any information about the actual application scene but instead uses the on-site tested data to train the symbolic regression model, and it features a high degree of localization accuracy and good method versatility.
(2) A high-sensitivity, high-precision, and low-noise electrostatic potential sensor was designed. For the first time, this sensor was used for the indoor location of moving people. The prototype was realized based on a commercial amplifier, which was capable of flexibly adjusting the sensor’s test distance, frequency response characteristics, power consumption, and production cycle according to the actual requirements.
(3) A prototype system was built using four sensors and the symbolic regression algorithm, where the results showed that the optimal model was able to achieve an 80% accuracy of the motion trajectory in a 5 m × 5 m test area, with a median error range of 0.4–0.6 m in terms of the absolute value.

The rest of this paper is organized as follows. Section 2 describes the principle of non-contact human electrostatic potential measurement. Section 3 describes the indoor personnel localization method and experimental design, electrostatic potential sensor, and symbolic regression machine learning method. Section 4 presents the experimental results of the indoor localization and discusses the factors that affected the results in detail. Finally, Section 5 concludes this paper and suggests directions for further work.

## 2. Principle

**Figure 1:**
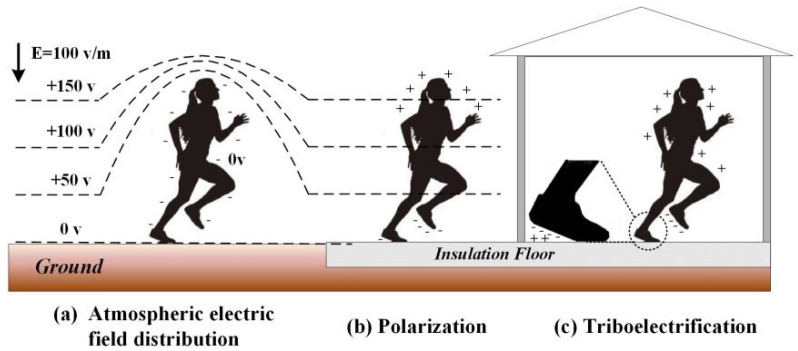
Principle of the electrostatic signal generated by moving the human body.

The physical mechanism of the electrostatic signal generated by a moving human body was discussed in detail in Feynman’s lecture on physics [31]. As shown in Figure 1, outside, there is an atmospheric electric field of about 100 V/m between the ionosphere and the earth.

**Figure 2:**
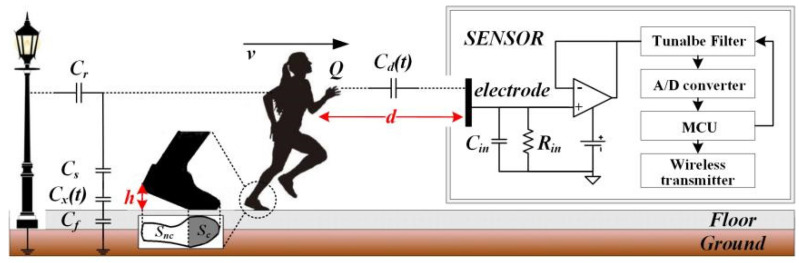
Test principle of the electrostatic potential of a moving human body.

The body will be polarized and charged when the human body is electrostatically insulated from the earth. Furthermore, when the human body is grounded, it distorts the vertical gradient of the atmospheric electric field in its vicinity, generating a measurable electrostatic signal. Indoors, the human body’s static electricity mainly comes from the rapid contact separation between body parts and objects, such as the friction between the sole of a shoe and the ground, the friction between an arm and a desktop, and the friction between clothes. The following is an example of human walking, as shown in Figure 2, to theoretically analyze the feasibility of this method.

As shown in Figure 2, the human body is an electrostatic conductor. There are two high-resistance layers between the body and the ground: the sole (usually rubber, EVA composite material, etc.) and the floor (wooden floor, tile, etc.). Therefore, the overall equivalent capacitance of the human body to the earth *C_B_* is

(1) $$C_{B}=C_{r}+\frac{1}{\frac{1}{C_{s}}+\frac{1}{C_{f}}+\frac{1}{C_{x}(t)}}$$

where *C_r_* is the coupling capacitance of a human body to the grounded objects in the surrounding environment (walls, streetlights, antistatic tables, metal carts, etc.), *C_s_* is the equivalent capacitance of shoe soles (including socks), and *C_f_* is the equivalent capacitance of the floor. During movement, the change in *C_s_* and *C_f_* is tiny and can be considered constant. *C_x_(t)* is the equivalent capacitance between the lower surface of the sole and the upper surface of the floor, which is a periodic function that is related to individual characteristics, such as step length, step frequency, posture, and motion category, where its expression is given in [32]. The electrostatic potential *U_B_* of a moving human body can be expressed as

(2) $$U_{B}=\frac{Q_{B}}{C_{B}}$$

*Q_B_* is the body’s charge and reference [33] expresses it. Thus, the induced charge *Q_s_* on the sensor measurement electrode at distance *d* can be expressed as

(3) $$Q_{s}=C_{d}\left(t\right)\left(U_{B}-V_{s}\right)$$

(4) $$C_{d}\left(t\right)=\frac{\varepsilon_{a}S_{e}}{d}=\frac{\varepsilon_{a}S_{e}}{D-f_{s}l_{B}t}$$

*C_d_(t)* is the equivalent capacitance between the human body and the induction electrode, which is determined by the equivalent area *S_e_* between the human body and the electrode, the step frequency *f_s_*_,_ and the step length *l_B_*. *V_s_* is the voltage on the induction electrode, which is much less than *U_B_*. Therefore, the sensor-induced current is

(5) $$I=\frac{dQ_{s}}{dt}=\frac{d\left(C_{d}\left(U_{B}-V_{S}\right)\right)}{dt}\approx \frac{d\left(C_{d}U_{B}\right)}{dt}=\frac{d\left(C_{d}\frac{Q_{B}}{C_{B}}\right)}{dt}=\frac{Q_{B}}{C_{B}}\cdot \frac{dC_{d}}{dt}+\frac{C_{d}}{C_{B}}\cdot \frac{dQ_{B}}{dt}-C_{d}\frac{Q_{B}}{C_{B}^{2}}\cdot \frac{dC_{B}}{dt}$$

The first term of (5) represents the induced current caused by the variation in the equivalent coupling capacitance *C_d_*, which is mainly influenced by the relative distance between the sensor and the human body. The second term represents the induced current caused by the change in *Q_B_*. According to [22], it is an exponential function that usually saturates after a few seconds to ten seconds of movement. Therefore, it contributes more to the induced current at the beginning and the end of the movement, while its contribution to the induced current during the motion is zero. The third term represents the change in the sensor-induced current caused by the change in *C_B_*, mainly the change in the equivalent capacitance of the human body to the ground and the change in the equivalent capacitance of the human body to the grounding body of the surrounding environment. Through the above theoretical analysis, it can be found that the electrostatic signal of a moving human body has strong specificity. It is not only influenced by the individual characteristic parameters of the moving human body but also by the distribution of the grounded body in the practical application environment. Indoor localization methods based on electrostatic signals need to overcome the impact of these factors on the accuracy of the results.

## 3. Method

### 3.1. Experimental Design

**Figure 3:**
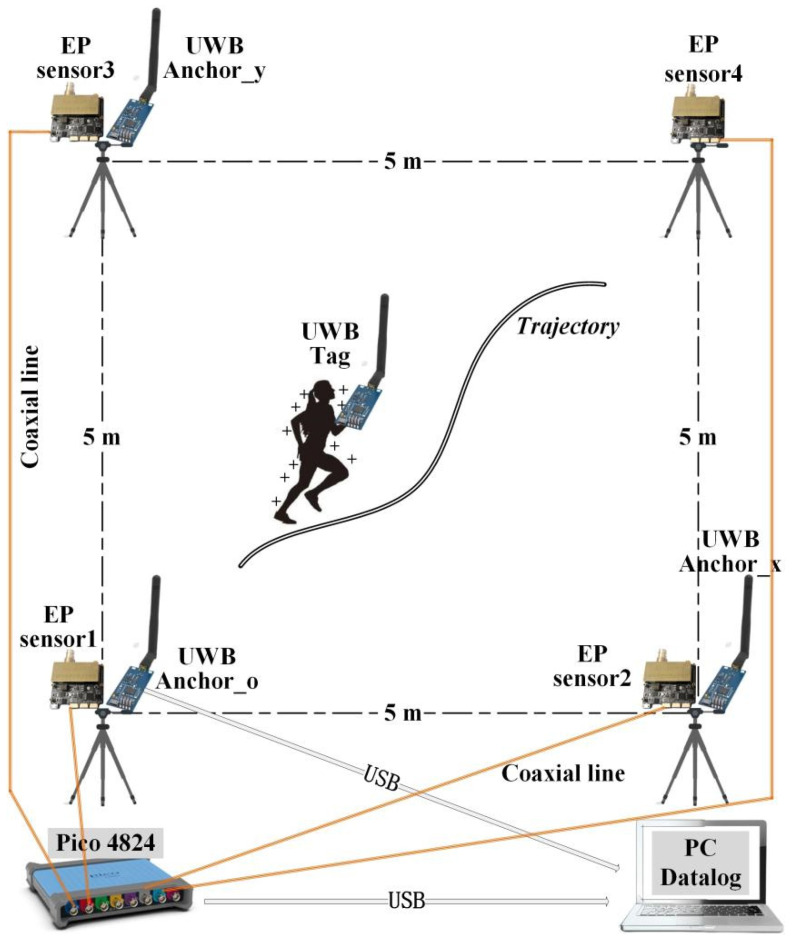
Experimental scene layout of the remote test of human electrostatic potential.

On the first floor of our laboratory, which has a width of 10 m and a length of 12 m, a square site with a tiled floor of 5 m × 5 m was selected. There was no grounding body at the test site, and the distance around the test site was not less than 1.5 m from the wall or off-site grounding body. The same electrostatic sensors were arranged at each of the four endpoints of the square test site to test the electrostatic potential of a moving human body in the test site. A coaxial cable connected the electrostatic sensor to a multi-channel digital storage oscilloscope (PicoScope 4824 from Pico, Hong Kong, China). The oscilloscope acquired the sensor output signal and transmitted it to a computer through a USB to complete the data recording. The experimental scene layout is shown in Figure 3.

The high-accuracy localization system adopted an ultra-wideband (UWB) electromagnetic pulse indoor localization system. It used the two-way time-of-flight method to obtain the distance from the human hand-held tag sensor to the base station sensor, combined the distance from the three base stations to the tag sensor, and used the trilateral measurement method to calculate the coordinates of the moving human body. One endpoint of the square test site (bottom-left endpoint in Figure 3) was defined as the coordinate origin, and the two adjacent endpoints were the X-axis endpoint and Y-axis endpoint. The base station sensors of the ultra-wideband positioning system (YCHIOT’s UWB Mini 3s Plus) were arranged on three endpoints (one sensor per endpoint) and placed on top of a 1.5 m high tripod; these endpoints were named UWB_Anchor_O, UWB_Anchor_X, and UWB_Anchor_Y. When the human moved with the ultra-wideband positioning tag sensor (UWB_Tag) in hand, the positioning system output the positioning result through UWB_Anchor_O with a refresh rate of 100 Hz. The data recording was completed by transferring the positioning data to a computer using a USB data cable. The spatial positioning accuracy of the ultra-wideband positioning system was 10 cm.

### 3.2. Electrostatic Potential Sensor Design

#### 3.2.1. The Principle of the Sensor Circuit

**Figure 4:**
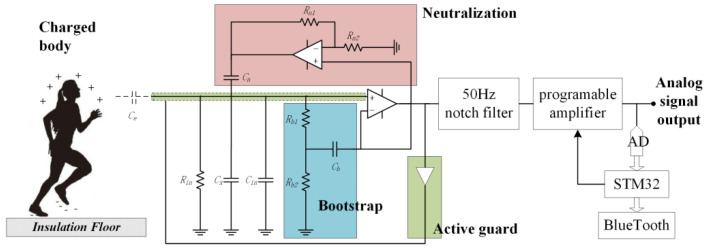
Circuit schematic of the electrostatic potential sensor.

The sensing electrodes of the electrostatic potential sensor and the measured human body were not in electrical or mechanical contact, and the distance between them was more than 1 m. Therefore, the equivalent coupling capacitance between the sensing electrodes and the measured human body was tiny. In addition, the measured human electrostatic potential signal had a low frequency and was susceptible to interference from 50 Hz/60 Hz power frequency signals. The electrostatic potential sensors needed to have a very small input capacitance and a very high input resistance to accurately detect the human electrostatic signal. Unlike the commercial electrostatic potential sensors EPIC used in the literature [1,2], this study comprehensively used three typical positive feedback electronic technologies of neutralization, a bootstrap, and an active guard, which were combined with shielding, a 50 Hz notch filter, and program-controlled amplification technology to design an electrostatic potential sensor. The principle is shown in Figure 4.

**Figure 5:**
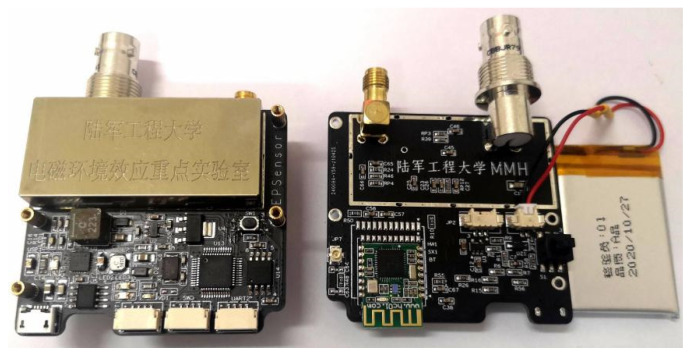
Electrostatic potential sensor.

As shown in Figure 4, *C_e_* is the equivalent capacitance coupling between the induction electrode and the charged body under test, which is determined by the diameter of the induction electrode and its distance to the authorized body. *C_in_*, *C_x,_* and *R_in_* are the input capacitance, stray capacitance, and input resistance of the amplifying circuit, respectively. *C_e_* forms a capacitive voltage divider network with *C_in_* and *C_x_*, while *C_e_* and *R_in_* form a first-order high-pass filter, both of which degraded the circuit’s sensitivity to amplifying the low-frequency electrostatic induction signal. The neutralizing circuit positively feeds the output signal to the input end through the neutralizing capacitance *C_n_* to reduce the equivalent input capacitance *C_in_*. The bootstrap and bias networks provide a controllable DC leakage channel to prevent saturation of the amplifier and reduce the leakage current at the positive input end of the amplifier in the low-frequency signal segment. The active guard drives the shielding case of the induction electrode using a unity gain amplifier to reduce the stray capacitance *C_x_*, thus maximally reducing the current leakage and power frequency interference. Compared with EPIC, the advantage of this sensor is that it can flexibly adjust the size of the sensing electrode and the frequency response characteristics of the circuit so that the test range and sensitivity can be flexibly adjusted. Moreover, it is possible to select amplifiers with different performance parameters according to different needs, thus meeting the requirements of a given practical application scenario regarding the sensor performance, power consumption, price, and production cycle. The electrostatic potential sensor was implemented using commercial discrete devices and a printed circuit board process, as shown in Figure 5.

The prototype sensor consists of a low-noise amplifier (ADA4530 from Analog device (Shanghai, China)), a power frequency notch filter circuit, an STM32 microcontroller, and an HC09 Bluetooth module packaged in a metal shielded box. By properly setting the resistance and capacitance values of the bootstrap and neutralization circuits, the equivalent input impedance of the sensor could reach a TΩ magnitude, with the input capacitance reaching a pF magnitude. It has analog and digital output ports and is capable of continuous measurement.

#### 3.2.2. Performance Test of the Sensor

**Figure 6:**
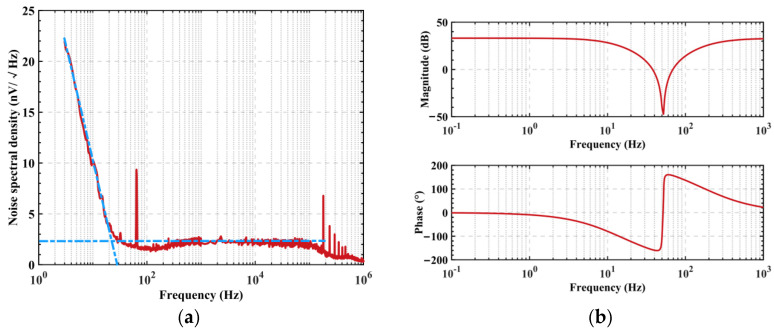
Circuit schematic of the electrostatic potential sensor. (**a**) Voltage noise spectral density. (**b**) Frequency response characteristic.

The noise spectral density of the sensor was tested in an unshielded environment using an Agilent E4440A spectrum analyzer. When the input port was grounded using a 1 pF capacitance, the output voltage noise spectral density shown in Figure 6a was produced. The 1/f corner frequency of the flicker noise was about 30 Hz, and the Gaussian white noise of the sensor when it was higher than this frequency point was 2.4 nV/√Hz, which determined the minimum detectable signal amplitude of the sensor. Under the same environment, the Digilent Analog-Discovery2 multi-function analyzer was used to analyze the frequency response characteristic of the sensor under the 1 pF coupling capacitance condition, with the curve shown in Figure 6b. Using the 50 Hz notch filter circuit, the voltage gain was about −30 dB near 50 Hz, which effectively decreased the power frequency interference. Furthermore, the voltage gain from 0.1 Hz to 1 kHz was 34 dB. It was capable of effectively amplifying the weak electrostatic induction signal.

### 3.3. Symbolic Regression Algorithm

Symbolic regression uses evolutionary algorithms to search the mathematical expression space to minimize the error between the measured data and the expression prediction data and automatically find the mathematical expression behind the measured data [34]. Unlike other linear or nonlinear regression methods, symbolic regression does not require a predefined form of the target expression. It can search both the form and parameters of the mathematical expression, significantly reducing the reliance on artificial prior knowledge and expanding its applicability, providing a flexible and simple method for nonlinear predictive modeling [35].

The advantages of the symbolic regression algorithm include: automatically creating concise and accurate equations for predicting the behavior of physical systems that are consistent with Occam’s razor; easy-to-deploy training to obtain symbolic models without the need for specialized hardware and software environments (such as neural networks and vector machines); and the model form is highly interpretable and concise, making it easier to revise than typical black box prediction models [36].

**Figure 7:**
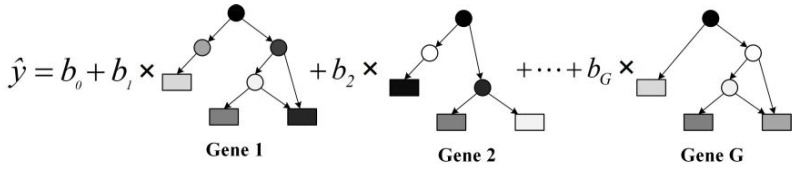
Schematic diagram of symbolic regression modeling.

This study used the multi-gene symbolic regression method, combined with the population search capability of multi-gene genetic programming (MGGP) and the parameter estimation capability of linear least squares, to find the optimal linear combination of mathematical expressions corresponding to all genetic individuals within a population and to minimize the error between the predicted output response of the mathematical model and the target output [25]. The principle of the method is shown in Figure 7.

In Figure 7, there are G gene individuals in the population, where each gene is a symbolic binary tree interpreted directly as a symbolic expression. Its leaf nodes are numerical constants, input or state variables, and other parameter variables. The middle nodes are mathematical operation symbols, including algebraic operators, such as addition, subtraction, multiplication, and division; trigonometric functions; exponential functions; Boolean functions; and other basic functions. Moreover, the output of the mathematical expression corresponding to each gene individual is $\hat{y_{1}}\ldots \hat{y_{G}}$, where their linear combination gives the output response prediction ŷ of the population mathematical model:

(6) $$\hat{y}=b_{0}+b_{1}\times {\hat{y}}_{1}+\ldots +b_{G}\times {\hat{y}}_{G}$$

where *b_0_* is the bias parameter and *b_1_*, …, *b_G_* are the scaling parameters. Since all input variables *x*, target output variables *y,* and response prediction *ŷ* are vectors of the same length, the above equation can be rewritten in matrix form as

(7) $$\hat{y}=G\times B$$

where *G* is the gene prediction response matrix and *B* is the linear combination parameter vector, which are

(8) $$G=\left[1\;{\hat{y}}_{1}\;{\hat{y}}_{2}\ldots \;{\hat{y}}_{G}\right]$$

(9) $$B={\left[b_{0}\;b_{1}\;\ldots \;b_{G}\right]}^{T}$$

The optimal value of *B* can be calculated from *y* and *G* using linear least squares estimation under several prerequisite assumptions, such as the independence and normal distributive property of the ***G*** column:

(10) $$B={\left(G^{T}G\right)}^{-1}G^{T}y$$

The expressions of primordial genetic individuals in a genetic population are formed by randomly combining basic mathematical units. Further, new expressions are generated using selection and cross-genetic operations. The fitness function was used to evaluate the fitting ability of all child expressions to the experimental data, and the best ones were retained. The above process was repeated until the expression satisfied the required accuracy or reached the optimization time (in this study, the optimization time was set to 24 h for a Lenovo workstation P700 with two INTEL Xeon E5-2620 V3 @2.4GHz processors and 52 GB RAM). The algorithm was terminated and the set of expressions most likely to represent the underlying mechanism of the data was returned. The fitness function was used to measure the fit of *ŷ* to *y*, which was calculated using the root-mean-square error (RMSE) in this study. The genetic operators included the following:

- Population initialization

For each individual of the primitive population, use the basic mathematical unit functions constant, input variable, addition, subtraction, multiplication, division, sine, cosine, exponential, and input variables to generate a symbolic binary tree with a depth limit, where the default range is 10. In the primary generation, it is important to ensure that there are no duplicate gene fragments between genetic individuals to enhance the diversity of the population.

- Selection operator

In the population, each individual participates in a Pareto tournament with its fitness and complexity as indicators to obtain its probability of being selected for genetic inheritance. Then, according to the probability value of each individual, it selects the candidate for the crossover operation.

- Crossover operator

Each parent randomly selects a gene, these genes are crossed in the subtree of a symbolic binomial tree, and the progeny genes replace the original genes in the parent model. Finally, a progeny is replicated in the new population.

## 4. Results and Discussion

### 4.1. Experimental Parameters

Three male participants were invited to participate in the experiment while wearing athletic shoes (rubber combined with EVA soles). Participants walked slowly (defined as walk) and ran rapidly (defined as run) ten times each at randomly selected starting points within the test site for each trial. The presence or absence of both feet off the ground simultaneously was used as the basis for differentiating walking from running. Sixty sets of data were obtained, including the participant’s positioning trajectory data during the experiment (time-series signals from three base station sensors) and human electrostatic potential test data (time series from four electrostatic sensors). The nomenclature of the test data was as follows: P1W represents ten sets of data recorded by the first subject who did a “walk” action ten times, P2R represents ten sets of data recorded by the second subject who did a “ran” action ten times, and so on. During the experiment, the air temperature at the site was 21 degrees Celsius and the relative humidity was 45%.

### 4.2. Data Cleaning and Alignment

**Figure 8:**
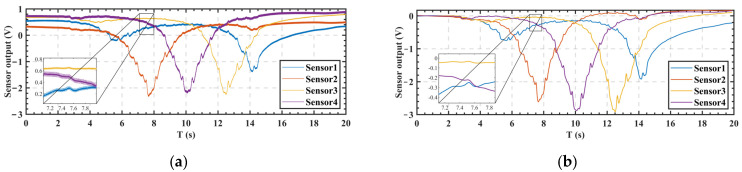
The output signals of the four electrostatic potential sensors. (**a**) Original data. (**b**) After data cleaning.

In the experiment, the output of the four electrostatic potential sensors was in the form of an analog signal that was synchronously recorded by the oscilloscope at a sampling rate of 50 KHz, as shown in Figure 8.

As seen in Figure 8a, the electrostatic signal during the human movement was a low-frequency signal below 1 Hz, and there was a 50 Hz power frequency noise and DC offset in the test results; this was due to the influence of the low-frequency electromagnetic radiation of the power supply line in the test field, the residual electrostatic potential of the human body, and the surrounding objects with static electricity. Therefore, data cleaning operations of filtering, down-sampling, and de-offset of the collected electrostatic potential data were required. The filtering operation used MATLAB’s low-pass digital filter function ‘lowpass ()’ to perform noise reduction on the recorded data, with the cutoff frequency parameter set to 1 Hz, the steepness parameter set to 0.9999, and the stopband attenuation parameter set to 1000 dB. The ‘resample ()’ function in MATLAB was selected for the down-sampling operation, the target sampling rate parameter was set to 100 Hz, and the sampling method was linear resampling. Finally, the DC offset was subtracted from the resampled data. The DC offset selected the mean value of the first 100 sample points of each data time series, which was the mean value of the electrostatic potential of the body of the tested human being standing still for 1 s before exercising. The results of the data cleaning are shown in Figure 8b.

**Figure 9:**
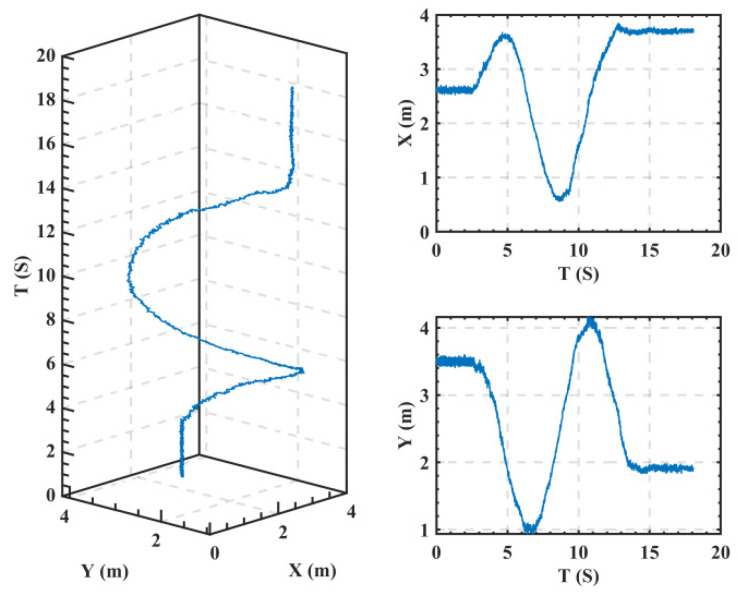
The test results of the moving body trajectory coordinates. The left picture gives the motion trajectory, the upper-right figure gives the *X*-coordinate, and the lower-right figure gives the *Y*-coordinate.

The sampling rate of the UWB positioning system was 100 Hz and the test results are shown in Figure 9.

Since the positioning error of this system was 0.1 m, the 0.1 m amplitude of the high-frequency noise could also be seen from the experimental data. Therefore, the ‘smoothdata ()’ function in MATLAB was used to smooth the original data to eliminate the noise in the positioning data, select the moving average method, and set the smoothing factor parameter to 0.05.

**Figure 10:**
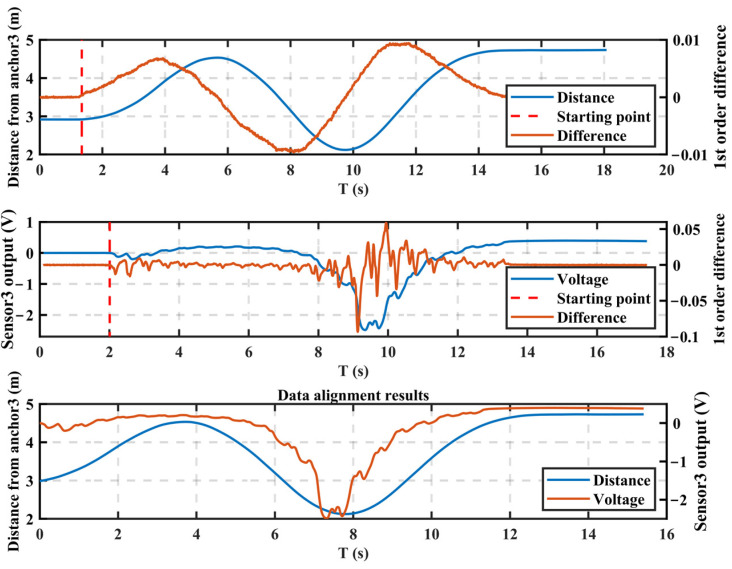
The alignment result of the electrostatic signal and the localization signal.

Two independent systems collected the human electrostatic signal and the localization signal. Neither system could temporally label the data sampling points; therefore, an alignment operation was required for these two data types. Before the test, the subject was allowed to stand still for two seconds, during which the two systems were activated. The exact synchronization of the activation time could not be guaranteed, but the data they recorded were kept constant. Subsequently, the subjects began to exercise and the recorded data from both systems produced fluctuations. This study used the data fluctuation point as the starting point flag for data alignment and performed alignment operations on the signals collected by the two systems. MATLAB’s ‘diff ()’ function was used to find the first-order difference values of the electrostatic signal and the positioning signal. The ‘ischange ()’ function was then used to find the first abrupt change in the differential signal, which was where the data alignment began. The minimum value of the starting point of the five-way electrostatic signal was used as the starting point moment of the electrostatic signal. The minimum value of the starting point of the three-way distance data was used as the starting point moment of the positioning signal. Before the starting point moment, the data samples were deleted from the respective original data. The data alignment operation was performed and the results before and after alignment are shown in Figure 10.

In Figure 10, the upper figure gives the detection result of the starting point in the distance data, which was from the third positioning anchor sensor; the central figure gives the starting point detection result of the test result of the third electrostatic potential sensor. The third positioning base station sensor and the third electrostatic potential sensor were in the same position; the lower figure gives the alignment result of the distance data and the electrostatic potential data.

### 4.3. Indoor Location Results

In this experiment, the test data of the electrostatic potential sensor was used as the input variable of the symbolic regression algorithm, and the coordinate trajectory data measured by the ultra-wideband positioning sensor was used as the target. It was expected that the coordinates of the moving human body would be inverted by monitoring the electrostatic potential. The original data collected by the sensor showed that the electrostatic signal contained more high-frequency components than the positioning signal. This was because the speed of each foot rising and falling took place faster than the body translation during the human movement. This signal difference obviously increased the difficulty of the localization inversion symbolic regression algorithm. To introduce this a priori knowledge into the modeling process, we pre-smoothed the electrostatically induced signal to eliminate its high-frequency components and used MATLAB’s ‘smoothdata ()’ function, choosing a moving average approach with the smoothing factor parameter set to 0.05.

The fitness function of the symbolic regression algorithm was the root-mean-square error. The normalized root-mean-square error (NRMSE) was chosen to verify the model’s validity. Because the range of motion and the absolute value of the electrostatic potentials varied significantly between different sets of data, the absolute root-mean-square error could not accurately assess the model’s accuracy.

(11) $$NRMSE=\frac{1}{y_{\max}-y_{\min}}\sqrt{\frac{\sum_{t=1}^{N}{\left({\hat{y}}_{t}-y_{t}\right)}^{2}}{N}}$$

**Figure 11:**
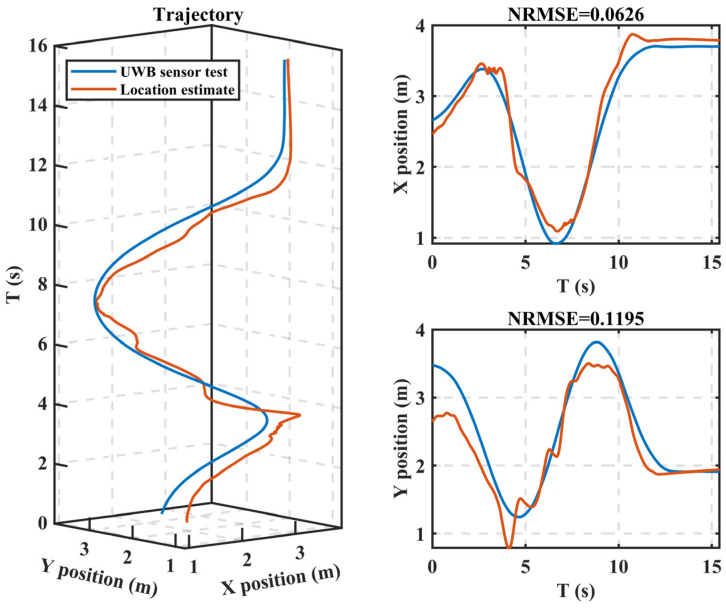
Typical results of the location inversion based on electrostatic potential signals. The left picture gives the motion trajectory, the upper-right figure gives the *X*-coordinate, and the bottom figure gives the *Y*-coordinate.

The “single-person single-set,” “single-person multi-set,” and “three-person multi-set” test data were selected as the training samples. The rest of the data were used for the validation samples. “Single-person single-set” means that only one set of test data of one person was selected. “Single-person multi-set” means that three sets of test data of one person were chosen. “Three-person multi-set” means that three sets of test data for each of the three persons were preferred. The data were randomly used for training in each experiment according to the above method. The initial values of the algorithm were randomly evolved while the other parameters remained unchanged, and the symbolic regression algorithm was run once to obtain a set of optimal models. The above process was repeated ten times. The optimal symbolic regression model was

(Model I) $$\begin{array}{l} X=0.677-\frac{2.58}{E_{s3}-1.47}-0.677sin\left(\frac{-3.07sin\left(E_{s2}-1.47E_{s1}\right)}{E_{s3}-1.47}\right)\\ -sin{\left(\frac{-2.58}{\left(E_{s3}-1.47\right)}\right)}^{2}sin\left(E_{s4}\right)sin\left(\frac{E_{s1}}{sin\left(E_{s4}\right)}+\frac{1.42}{1.42-E_{s2}}-E_{s1}\right) \end{array}$$

(Model II) $$\begin{array}{l} Y=2.13+E_{s1}+0.288E_{s2}+2.13E_{s4}E_{s1}{}^{2}-sin(E_{s3})-0.7E_{s4}\\ +0.351cos(8.51sin(E_{s1})-E_{s3}-0.755E_{s4}-2.13E_{s2}) \end{array}$$

where *E_s_*_1_*–E_s_*_4_ represent the corresponding electrostatic potential sensor outputs and models I and II were derived from the “multi-person, multi-set” experiment; the inverse localization results of the models on the P3W7 data sample are shown in Figure 11.

**Figure 12:**
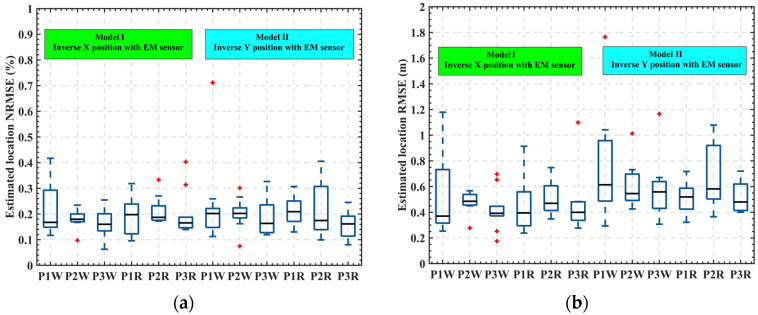
Error statistics results: (**a**) NRMSE statistics and (**b**) RMSE statistics.

In Figure 11, the NRMSE of the model was 0.0626 for the *X*-coordinate inversion result and 0.1195 for the *Y*-coordinate inversion result. The model’s errors for all of the data are shown in Figure 12a, where the median NRMSE for all the data sets was lower than 0.20, indicating that the model could achieve 80% accuracy in inverting the motion trajectory in the 5 m × 5 m test area. While the absolute value of the inverse motion trajectory error is shown in Figure 12b, the median range of the RMSE was 0.4–0.6 m and we believe that the error mainly came from the testing process.

During the test, the moving human held the UWB positioning tag sensor (size 0.01 m × 0.02 m × 0.1 m) fixed on one side of the body with one hand, randomly selected the starting point, and moved freely in the test site. In the process of moving, the orientation of the human body at a particular position was bound to be somewhat different or even opposite, which could make the human coordinate data obtained from the UWB positioning system test pathological, that is, the same human position corresponded to multiple different coordinate data. Assuming that the width of the human body was 0.6 m, the tag sensor could shift to the other body orientation, and the resulting positioning error could reach 0.6 m. The induction electrode of the electrostatic potential sensor belongs to an “omnidirectional antenna,” which senses the electrostatic field of the human body, which is mainly affected by the position of the human body and is hardly affected by the body’s orientation. Therefore, the model’s accuracy obtained using the symbolic regression algorithm was expected to improve further when the pathological localization data were eliminated. In addition, this experiment used four sensors to realize the positioning inversion in the space of 5 m × 5 m, which had a larger test range than previous literature and was responsible for the increased error. In the future, the error of positioning inversion is expected to reduce by further adjusting the size of the sensor induction electrodes.

### 4.4. Practicality and Limitations

The following advantages of this method were demonstrated by the experimental results. First, the localization accuracy of this method could reach 0.4–0.6 m. This was comparable to the typical breadth of a human shoulder and completely satisfied the need for human localization. Second, a circuit structure of the electrostatic potential sensor was proposed in this study. The hardware cost mainly came from the commercial amplifier chip in it. It is possible to select amplifiers with different performance parameters that meet the application requirements for sensor performance, power consumption, price, and production cycle. When choosing a common commercial amplifier chip, the cost of this sensor will be comparable to that of a BLE device. Third, the symbolic regression algorithm required very little processing power compared with the current popular deep learning methods. In the model-training phase, an ordinary desktop computer was required to run for one day to obtain a satisfactory model. The resulting model was a collection of mathematical formulas built on the fundamental unit functions of mathematics, including constant, input variable, addition, subtraction, multiplication, division, sine, cosine, exponential, and input variables. Additionally, the trained symbolic models could be deployed without requiring specialized hardware or software environments (such as neural networks and vector machines). Therefore, the computational complexity of the model is quite low. A common embedded processor (such as STM32 F103) can implement the real-time application of people’s movement tracking. Fourth, this method does not require the user to carry any additional devices. The system does not require regular calibration and other routine maintenance. Fifth, this method only collects the electrostatic field information of the moving human body. It will not collect any additional information involving personal privacy. Sixth, this method is passive and does not actively emit any signal. Therefore, this method has the features of high positioning accuracy, low system power consumption, low hardware cost, low algorithm complexity, high user convenience, good privacy protection, and a friendly environment.

In practical applications, the system requires an initial calibration after it has been set up. The initial calibration requires a small amount of time, less than one hour for data collection and less than one day for model training. The entire process requires no qualified staff. It will, however, involve forbidding activities in the area during data acquisition. This is due to the symbolic regression algorithm requiring few training samples and computational resources. The experimental results described in the study were acquired by training models on a personal computer over one day by utilizing sixty motion trajectory data. The outcomes of the experiment were satisfactory and reproducible over several trials. After the initial calibration, a recalibration will not be required if the system arrangement is not changed.

In addition, more problems need to be solved to enhance the usefulness of this method. First, there is the issue of distinguishing between several occupants. When multiple individuals are present in the same testing field, their electrostatic fields will be overlaid and measured by the same sensor. The mixed electrostatic induction signal will significantly decrease the localization accuracy. To address this problem, we need to add a signal separation process before the symbolic regression algorithm. According to the results of the theoretical analysis in Section 2, the electrostatic induction signal was related to the individual motion characteristics parameters, including the step frequency and the step length parameters. In the next step, we will use this information for the signal separation algorithm. The second issue is the influence of clothing, shoes, and flooring materials. They can drastically alter the amount of electrostatic charge and electrical potential of the human body. This affects the signal amplitude of the sensor, and thus, mainly affects the testing range and accuracy of this system. In the future, we will address this issue by increasing the size of the sensing electrodes and the sensor density. Third, the electronic products in the site will not generate interference as the electrostatic signal is directly related to the human body movements. It is primarily a signal with a frequency below 50 Hz. Therefore, the sensor’s interference signal is mostly the power frequency signal from the power line rather than the high-frequency signal emitted by electronic products. The sensor has a power frequency notch filter circuit built into its design. Consequently, the electromagnetic radiation signal of electronic products will not impact the system’s performance.

The limitation of this method is that it relies on the electrostatic signal of a moving individual. The generation of electrostatic signals from the human body is governed by the leakage resistance of the human body to the ground. Factors such as the clothing and footwear of a person, the air humidity of the environment, the floor material, and the floor all affect a human body’s leakage resistance. In some special cases, a human body will barely generate electrostatic potential, thus making this method ineffective, for example, in climatic conditions where the air humidity is particularly high, in an anti-static work area, when the human body is connected to the anti-static wristband, or when a static ion fan is deployed. In addition, the electrostatic field of the human body will be well-grounded when using wall shielding such that this method can not be used through a wall.

## 5. Conclusions

Based on the symbolic regression machine learning method, this study proposed an indoor localization method; designed a high-sensitivity, high-precision, and low-noise electrostatic potential sensor; and built a prototype test system to identify the indoor location of a moving human body. This method obtains the transfer function from the electrostatic sensor signal to the location coordinates on site without requiring physical information about the actual scene. The prototype test system is simple and practical; adaptable and easy to integrate; and has a better balance of positioning accuracy, system power consumption, privacy protection, and environmental friendliness.

Regarding future work, first, the realization of the simultaneous detection of multiple individuals is the primary issue for the practical application of this method. Theoretical analysis demonstrated that the electrostatic induction signal contains the motion characteristic parameters of individuals. A multi-target detection method based on feature recognition and multi-sensor data fusion techniques is feasible. Second, the human motion parameters obtained from the electrostatic signal can be equally used for the prevention and diagnosis of human motion-related diseases, which will be of great value in elderly care, such as the diagnosis of Alzheimer’s disease based on an electrostatic signal [32]. Third, further test scenarios are required to examine the stability of this system under various environmental conditions, such as the site, floor material, floor, person, clothing, footwear, temperature and humidity, and climate. Fourth, attempts can be made to integrate this system with mature technologies, such as smart building control systems, 5G communication, and AI IoT to form a more easily accessible and usable hardware system.

## Figures and Tables

**Table 1:** Comparison of the indoor localization methods.

Category	Method	Localization Accuracy	Power Consumption	Hardware Cost	Algorithm Complexity	User Convenience	Privacy Protection	Environmental Friendliness
Tag -based	WIFI [7,8,9]	High√	High	High	High	Low	High√	Low
	BLE [10]	Low	Low√	Low√	High	Low	High√	Low
	RFID [11]	Low	Low√	Low√	Low√	Low	High√	Low
	UWB [12]	High√	High	High	High	Low	High√	Low
	LIFI [13]	High√	High	High	High	Low	Low	High√
Tag-free	Gas [14]	Low	Low√	Low√	Low√	High√	High√	High√
	Sound [15]	Low	Low√	Low√	Low√	High√	Low	High√
	Vibration [16]	Low	Low√	Low√	Low√	High√	High√	High√
	Heat radiation [17]	Low	Low√	Low√	Low√	High√	High√	High√
	Light reflection [18]	High√	High	High	High	High√	Low	High√
	RF signal reflection [19]	High√	High	High	High	High√	High√	High√
Tag-free	**Electrostatic** **(this work)**	High√	Low√	Low√	Low√	High√	High√	High√

√ represents the desired goal.

## Data Availability

The study did not report any data.
